# The Longitudinal Youth in Transition Study (LYiTS) Cohort Profile: Exploration by Hospital- Versus Community-Based Mental Health Services

**DOI:** 10.1177/07067437221115947

**Published:** 2022-08-04

**Authors:** Kristin Cleverley, Julia Davies, Sarah Brennenstuhl, Kathryn J. Bennett, Amy Cheung, Joanna Henderson, Daphne J. Korczak, Paul Kurdyak, Andrea Levinson, Antonio Pignatiello, Katye Stevens, Aristotle N. Voineskos, Peter Szatmari

**Affiliations:** 1 Lawrence S. Bloomberg Faculty of Nursing, 7938 University of Toronto, Toronto, ON, Canada; 2 7978 Centre for Addiction and Mental Health, Toronto, ON, Canada; 3 Department of Psychiatry, Temerty Faculty of Medicine, 7938 University of Toronto, Toronto, ON, Canada; 4 Health Research Methods, Evidence and Impact (formerly Clinical Epidemiology and Biostatistics), 62703 McMaster University Faculty of Health Sciences, Hamilton, ON, Canada; 5 71545 Sunnybrook Health Sciences Centre, Toronto, ON, Canada; 6 Department of Psychiatry, 7979 Hospital for Sick Children, Toronto, ON, Canada

**Keywords:** mental health, youth, adolescent psychiatry, cohort profile, latent profile analysis

## Abstract

**Objectives:**

Youth face numerous challenges in receiving coordinated and continuous mental health services, particularly as they reach the age of transition from child and adolescent mental health services (CAMHS) to adult mental health services (AMHS). The Longitudinal Youth in Transition Study (LYiTS) follows youth prospectively as they cross this transition boundary to better understand their transition pathways and resulting symptoms and health service use outcomes. The current paper presents the baseline profile description for the LYiTS cohort and additionally examines differences in symptoms and functioning and health service utilization between youth receiving services at hospital- versus community-based CAMHS.

**Methods:**

A cross-sectional design was used. A sample of 237 16–18-year-old youth recruited from outpatient CAMHS at two hospitals and two community sites completed self-report measures at their first of four annual assessments. A latent profile analysis was conducted to identify symptomology profiles, and youth were compared on symptoms and health service use between hospital- and community-based sites.

**Results:**

Four distinct symptomology profiles were identified (subclinical, moderate internalizing, moderate externalizing, and high symptomology). Symptom profiles and functioning levels reported by youth were no different across both types of organization, although there were differences detected in health service utilization, such as type of provider seen and use of medications.

**Conclusions:**

These findings suggest that there is little difference in symptomology between youth accessing hospital versus community-based CAMHS. With growing interest in understanding the effectiveness and cost-effectiveness of different models of mental health care, these findings provide a new understanding of the clinical and service use profiles of transition-aged youth that will be explored further as this cohort is followed across the CAMHS to AMHS transition boundary.

## Abrégé

**Objectifs:**

Les jeunes rencontrent de nombreux problèmes pour recevoir des services de santé mentale coordonnés et continus, particulièrement quand ils atteignent l'âge de transition des services de santé mentale pour enfants et adolescents (SSMEA) aux services de santé mentale pour adultes (SSMA). L'Étude longitudinale sur les jeunes en transition (ELJT) suit les jeunes prospectivement quand ils croisent la limite de cette transition pour mieux comprendre les trajectoires de leur transition et les symptômes en résultant ainsi que les résultats sur l'utilisation des services de santé. Le présent article montre la description du profil de base pour la cohorte de l'ELJT, et examine en outre les différences de symptômes et de fonctionnement et de l'utilisation de services de santé entre les jeunes recevant des services à l'hôpital contre des SSMEA en milieu communautaire.

**Méthodes:**

Une conception transversale a été utilisée. Un échantillon de 237 jeunes de 16 à 18 ans recrutés parmi des patients ambulatoires SSMEA de deux hôpitaux et de deux sites communautaires ont répondu aux mesures d'auto-déclaration à leur première de quatre évaluations annuelles. Une analyse de profil latent a été menée pour identifier les profils de symptomatologie, et les jeunes ont été comparés selon les symptômes et l'utilisation des services de santé entre les hôpitaux et les sites communautaires.

**Résultats:**

Quatre profils distincts de symptomatologie ont été identifiés (sous-clinique, internalisant modéré, externalisant modéré, et symptomatologie élevée). Les profils de symptômes et les niveaux de fonctionnement rapportés par les jeunes n'étaient pas différents dans les deux types d'organisations, bien que des différences aient été détectées dans l'utilisation des services de santé, comme le type de prestataire vu et l'usage des médicaments.

**Conclusion:**

Ces résultats suggèrent qu'il y a peu de différences de symptomatologie entre les jeunes ayant accès à l'hôpital contre ceux qui reçoivent des SSMEA communautaires. Vu l'intérêt croissant pour comprendre l'efficacité et la rentabilité de différents modèles de soins de santé mentale, ces résultats procurent une nouvelle compréhension des profils cliniques et d'utilisation des services des jeunes en âge de transition qui feront l'objet de plus d'exploration lorsque cette cohorte sera suivie à la frontière de la transition entre SSMEA et SSMA.

## Introduction

The transition from childhood to adulthood represents a period of vulnerability to poor mental health and functioning. Transition-aged youth (TAY), those approximately 16–24 years old,^1^ experience multiple developmental milestones: engaging in post-secondary education, entering the workforce, and moving out of their family/guardians’ home. These milestones, characterized by increasing autonomy, occur during a high-risk period for the onset of mental or substance use disorders.^1,2^ Against this background, youth commonly face a service boundary at age 18,^3–6^ when they age out of child and adolescent mental health services (CAMHS) and must transition to adult mental health services (AMHS).

Although many youth approaching the age limit for CAMHS continue to have care needs,^3,7^ transition from CAMHS to AMHS is poorly coordinated.^3,7–9^ Consequently, a significant drop in service engagement occurs at this time.^10^ Studies have reported up to 60% of youth experience discontinuities in mental health care at age 18.^8,11^ Youth describe the experience of transitions between CAMHS and AMHS as disconnected and confusing.^12^ In Ontario, there was a 102% increase in mental health and substance-related emergency department (ED) visits among 18–20 year-olds between 2006 and 2017,^13^ suggesting needs are not being met across the age of transition. Youth with mental illness who experience fragmented care during this time are more likely to require ED visits and hospital admissions.^14^ However, little is known about continuity of mental health care across the transition boundary and its impact on youths’ symptoms and functioning.^15^

Understanding the health care contexts within which transitions occur will help to tailor interventions. CAMHS can be accessed in both hospital- and community-based settings. While hospital-based services typically focus on acute stabilization, medication management and psychosocial treatments, community-based services emphasize strengths-based, recovery-oriented services accessible to local populations and engage a wide network of supports and resources.^16–18^ In Ontario, access to community CAMHS is usually through self-referral, while access to hospital-based CAMHS often requires physician referral or care pathways initiated in the ED. Hospital- and community-based CAMHS were, until recently, funded through separate government ministries, with poor inter-ministry communication.^19^ While pathways into care can differ for hospital- and community-based CAMHS,^20^ youth often seek community-based care and physician services concurrently.^21^ This is expected given the numerous access points for mental health care^20,22^ and a system that often lacks a common/uniform intake process.^19^ Given the lack of systems coordination, it is not known if there are clinical differences between youth who end up receiving care in hospital- versus community-based CAMHS.

The Longitudinal Youth in Transition Study (LYiTS)^23^ is a prospective longitudinal cohort study of TAY accessing hospital and community-based CAMHS prior to transition to AMHS. Using data from the LYiTS cohort, the objectives of this study are to (1) understand differences in (i) symptoms and functioning and (ii) health and health care use among youth accessing CAMHS programmes in hospital compared to the community; and (2) investigate heterogeneity in mental health symptom presentation. We hypothesize that due to focus on high acuity and stabilization, hospital-based services will contain a greater proportion of youth exhibiting more severe symptom profiles. Understanding whether differences exist between youth accessing hospital- and community-based CAMHS will provide insight into whether youth are receiving services aligned with their acuity and needs, and ensuring interventions are designed to support effective pathways into and through the mental health care system.

## Methods

### Study Design

Data are derived from the LYiTS cohort study of youth aged 16-18 who received CAMHS and reach the CAMHS/AMHS transition boundary. The sample consists of clinically-referred youth receiving outpatient treatment in one of four CAMHS clinics in Toronto, Canada. The data reported represent the baseline measurement of the cohort from June 2016 to March 2020. The paper adhered to the STROBE reporting guideline^24^ (see Supplemental Table). The study received research ethics board approval at the Centre for Addiction and Mental Health (CAMH), at the Hospital for Sick Children (SickKids), George Hull Centre (GHC), and the University of Toronto. Youth with lived experience of CAMHS, clinicians and administrators from all sites were engaged throughout the study design and recruitment, as described in detail within the protocol.^23^

### Setting

Recruitment occurred from two academically-affiliated hospitals and two community CAMHS. The hospitals included CAMH, a psychiatric hospital for all ages, and SickKids, a paediatric hospital, both located centrally. At CAMH, participants were recruited from the child and youth outpatient service, and at SickKids from ambulatory and urgent care psychiatry services. Both services include mainly mood and/or anxiety disorders; access to services is through physician referral or via the ED. The community sites included GHC, and SickKids Centre for Community Mental Health (CCMH), both academically affiliated teaching sites provide CAMHS for multiple diagnoses up to the age of 18. Participants were recruited from day treatment, outpatient, and school-based programmes; access to services is through self-referral. All services are publicly funded. Clinicians and administrators from all sites were engaged in the co-design of the initial protocol, providing input into research questions, recruitment, study concepts and measures.

### Population

Participants were included in the cohort if they: (i) were aged 16 to 18 years; (ii) were receiving outpatient treatment in a CAMHS at the time of recruitment and (iii) could speak and read English. All youth listed as an active patient in health records and/or had scheduled appointment during the recruitment period were screened for eligibility. Further details of the cohort design are reported in the study protocol.^23^ The planned sample size was 350^23^; however, due to coronavirus disease 2019 (COVID-19)-related barriers, baseline recruitment that began in June 2016 was halted in March 2020 after determining that sample size requirements for the analysis of the primary objectives were met.

### Procedure

Participants provided written informed consent and completed self-report study measures in person on paper or electronically using the REDcap system over a period of approximately 45–90 min. Participants were provided an honorarium of $30.

### Measures

#### Organization Type

Organizational type was determined by the programme being accessed for CAMHS (i.e. hospital- or community-based programme). The length of time of use (in years) of the primary programme was self-reported.

#### Background Characteristics

Sample demographics included sex assigned at birth, gender identity, sexual orientation, age, cultural group identity, living situation, enrolment in school and employment status.

### Outcomes

#### Symptoms and Functioning

The Youth Self Report (YSR)^25^ is a valid and reliable youth self-report tool containing 119 items assessing eight empirically derived syndromes: anxious-depressed, withdrawn-depressed, somatic complaints, social problems, thought problems, attention problems, rule-breaking behaviour and aggressive behaviour. Internal consistency for the scales in this sample ranged from α = 0.67 (social problems) to α = 0.85 (anxious-depressed). To provide population standardization, raw summary scores for each syndrome scale can be expressed as *T* scores. *T*-scores of ≥70 can be interpreted as clinically elevated.^25^

To identify high-risk alcohol disorders and other drug use problem behaviours, the CAGE-Adapted to Include Drugs (CAGE-AID) questionnaire was used.^26^ This 4-item scale is summed to a possible total score of 4; a score of ≥1 indicates a possible drug or alcohol problem. In this sample, internal consistency was α = 0.82.

The 13-item Columbia Impairment Scale (CIS)^27^ was used to measure global functional impairment. Higher scores indicate poorer functioning and a score of ≥16 indicates more severe functional impairment.^28^ Participants can indicate that items are non-applicable therefore individual mean imputation was used when calculating the total score.^29^ The internal consistency was α = 0.81 in this sample.

#### Health and health care use

Presence of current health conditions was assessed using a study-specific checklist of 40 common conditions (e.g., cancer, asthma). The five most prevalent conditions and presence of multimorbidity, defined as having ≥2 conditions in addition to the mental health condition, are reported.

Youth completed the Health and Social Service Utilization measure, adapted from Browne et al.,^30,31^ a structured interview measuring past 6-month use of services billable to publicly funded health insurance (physician visits, ED visits) and non-billable (private therapist, drop-in agencies, online counseling, etc.) We report on access to a family doctor, psychiatrist use, use of other mental health providers (including social workers, psychologists), ED use, hospital admissions and any medication use.

### Statistical Analysis

The sample was summarized using descriptive statistics.

*Objective 1. YSR.*

*T* scores were calculated from the raw syndrome scale scores and described using means and standard deviations. The percentage of those with clinically-elevated symptoms, defined as with a *T* score of ≥70^25^ was tabulated and compared across groups using the Chi-Square test. The mean CIS score was calculated and compared across organizational types using an independent *t*-test (adjusted for unequal variance when required). The percentages scoring above each of the CIS and CAGE cut offs were calculated and compared across groups using the Chi-Square test. To investigate heterogeneity in the presentation of symptoms based on the total scores of the eight YSR syndrome scales, Latent Profile Analysis (LPA) was undertaken. Details of the LPA analytic method are provided in the Supplemental File (available online). Mean CIS and CAGE scores also compared across the obtained profiles using one-way analysis of variance.

*Objective 2*. The proportions of (i) having a family doctor, (ii) psychiatrist use, (iii) use of psychiatrist as the primary care, provider (iv) use of allied health care professionals, (v) ED use, (vi) any hospital admission, (vii) medication use in the past 2 days; (viii) health conditions and (ix) multimorbidity were compared between groups using the Chi-Square test. Median number of psychiatrist visits, allied health care professional visits, ED visits, hospital admissions and hospital days was calculated with the interquartile range (IQR) and compared across groups using the Mann–Whitney test.

There was minimal missing data. Where missing data was present, a complete case analysis was used. All tests were two-sided. Due to the large number of comparisons undertaken, statistical significance was established as p < .01 to reduce type II errors. All analyses were undertaken in SAS(v9.4) unless otherwise indicated.

## Results

### Eligibility and Recruitment

A total of 435 youth were confirmed to meet eligibility criteria. Of these, 35 declined to participate prior to consent, 150 were agreeable to discuss the study, but could not be contacted, or were no longer enrolled in services, 10 were determined ineligible, and 3 provided consent but did not complete the baseline assessment. A total of 237 confirmed eligible youth completed the informed consent process and provided baseline data. Of these, 137 were receiving treatment in hospital (57.8%) and 100 in the community (42.2%). Youth used CAMHS at their primary organization for a median of 1 year (IQR = 0–2 range 0–11); There was no difference in central tendency according to organizational type (*p* = 0.25); the range was also similar by setting type (hospital:0–10; community:0–11).

As shown in Table 1, there were no significant differences by organizational type for most demographics. The largest share of the sample was aged 17 (46.6%). More of those receiving care in the hospital were aged 18 than in the community (20.4% vs. 7.1%, *p* = 0.01).

**Table 1.** Demographic Characteristics of the Longitudinal Youth in Transition Study (LYiTS) Cohort.

	Overall (*n* = 237)	Hospital (*n* = 137)	Community (*n* = 100)	*p* value
	*n* (%)	*n* (%)	*n* (%)	
Sex assigned at birth				
Female	169 (71.3)	97 (70.8)	72 (72.0)	0.841
Male	68 (28.7)	40 (29.2)	28 (28.0)	
Gender identity				
CIS woman	148 (62.5)	83 (60.6)	65 (65.0)	0.69
CIS man	66 (27.9)	39 (28.5)	27 (27.0)	
Another identity (e.g. non-binary, trans, gender fluid)	23 (9.7)	15 (11.0)	8 (8.1)	
Sexual orientation				
Straight/heterosexual	120 (50.6)	74 (54.0)	46 (46.0)	0.142
Bisexual	57(24.1)	28 (20.4)	29 (29.0)	
Lesbian/Gay/Queer	22 (9.3)	16 (11.7)	6 (6.0)	
Another identity not already listed^a^	38 (16.0)	19 (13.9)	19 (19.0)	
Age				
'16	92 (38.8)	52 (38.0)	40 (40.0)	0.013
'17	110 (46.6)	57 (41.6)	53 (53.0)	
'18	35 (14.8)	28 (20.4)	7 (7.0)	
Cultural group identity				
white (Canadian, European)	119 (51.3)	69 (51.1)	50 (51.6)	0.364
Mixed	40 (17.2)	20 (14.8)	20 (20.6)	
Asian	26 (11.2)	19 (14.1)	7 (7.2)	
Black (Canadian, African, Caribbean)	12 (5.2)	5 (3.7)	7 (7.2)	
Latin American	14 (6.0)	8 (5.9)	6 (6.2)	
Another identity not already listed^a^	21 (9.1)	14 (10.4)	7 (7.2)	
Current living situation				
With parent(s)/family home	218 (92.4)	128 (93.4)	90 (90.9)	0.471
Not in family home	18 (7.6)	9 (6.6)	9 (9.1)	
Currently enrolled in school				
Yes	219 (92.4)	123 (89.8)	96 (96.0)	0.074
No	18 (7.6)	14 (10.2)	4 (4.0)	
Any work for pay in past 4 weeks				
Yes	127 (53.6)	67 (48.9)	60 (60.0)	0.091
No	110 (46.4)	70 (51.1)	40 (40.0)	

^a^ Categories had to be collapsed to maintain anonymity of the data.

The percentage with clinically elevated *T* scores was not different by organization type for any YSR syndrome scale (See Table 2). The scales with the most scoring ≥70 for both groups were Anxious-Depressed (48.1%) and Withdrawn-Depressed (33.3%).

**Table 2.** YSR Syndrome Scale T-Scores and Comparison of Proportion of Participants With T-Scores Over 70 Between LYiTS Compared by Hospital- Versus Community-Based CAMHS.

YSR Syndrome Scale	Total cohort *N* = 237			Hospital *N* = 137			Community *N* = 100			*p* value for comparison of % scoring >70 between hospital and community CAMHS
	Total *N*	Mean score (SD)	*n* scoring >70 (%)	*N*	Mean score (SD)	*n* scoring >70 (%)	*N*	Mean score (SD)	*n* scoring > 70 (%)	
Anxious-depressed	233	70.72 (11.80)	112 (48.1)	135	70.76 (11.75)	66 (48.9)	98	70.61 (11.88)	46 (46.9)	0.769
Withdrawn-depressed	234	67.64 (10.54)	78 (33.3)	135	67.12 (9.85)	41 (30.4)	99	68.21 (11.46)	37 (37.4)	0.262
Somatic complaints	236	63.56 (10.10)	55 (23.3)	136	63.06 (9.70)	27 (19.9)	100	64.10 (10.67)	28 (28.0)	0.145
Social problems	234	63.78 (8.74)	55 (23.5)	135	63.50 (8.45)	27 (20.0)	99	64.15 (9.11)	28 (28.3)	0.140
Thought problems	230	64.66 (9.03)	56 (24.4)	132	64.98 (9.16)	35 (26.5)	98	64.15 (8.86)	21 (21.4)	0.374
Attention problems	237	65.62 (11.30)	68 (28.7)	137	65.69 (11.90)	40 (29.2)	100	65.39 (10.49)	28 (28.0)	0.841
Rule-breaking behaviour	231	60.98 (7.87)	25 (10.8)	133	60.10 (7.16)	13 (9.8)	98	62.21 (8.59)	12 (12.2)	0.550
Aggressive behaviour	236	57.61 (7.51)	17 (7.2)	136	57.38 (7.39)	10 (7.4)	99	57.93 (7.67)	7 (7.0)	0.918

SD = Standard deviation; CAMHS = child and adolescent mental health services; LYiTS = Longitudinal Youth in Transition Study; YSR = Youth Self Report.

The LPA indicated distinct symptom profiles based on model fit indices. Details of the modelling can be found in the Supplemental File. Several model fit indices pointed to the four-profile model, which was ultimately selected.

**Figure 1.**
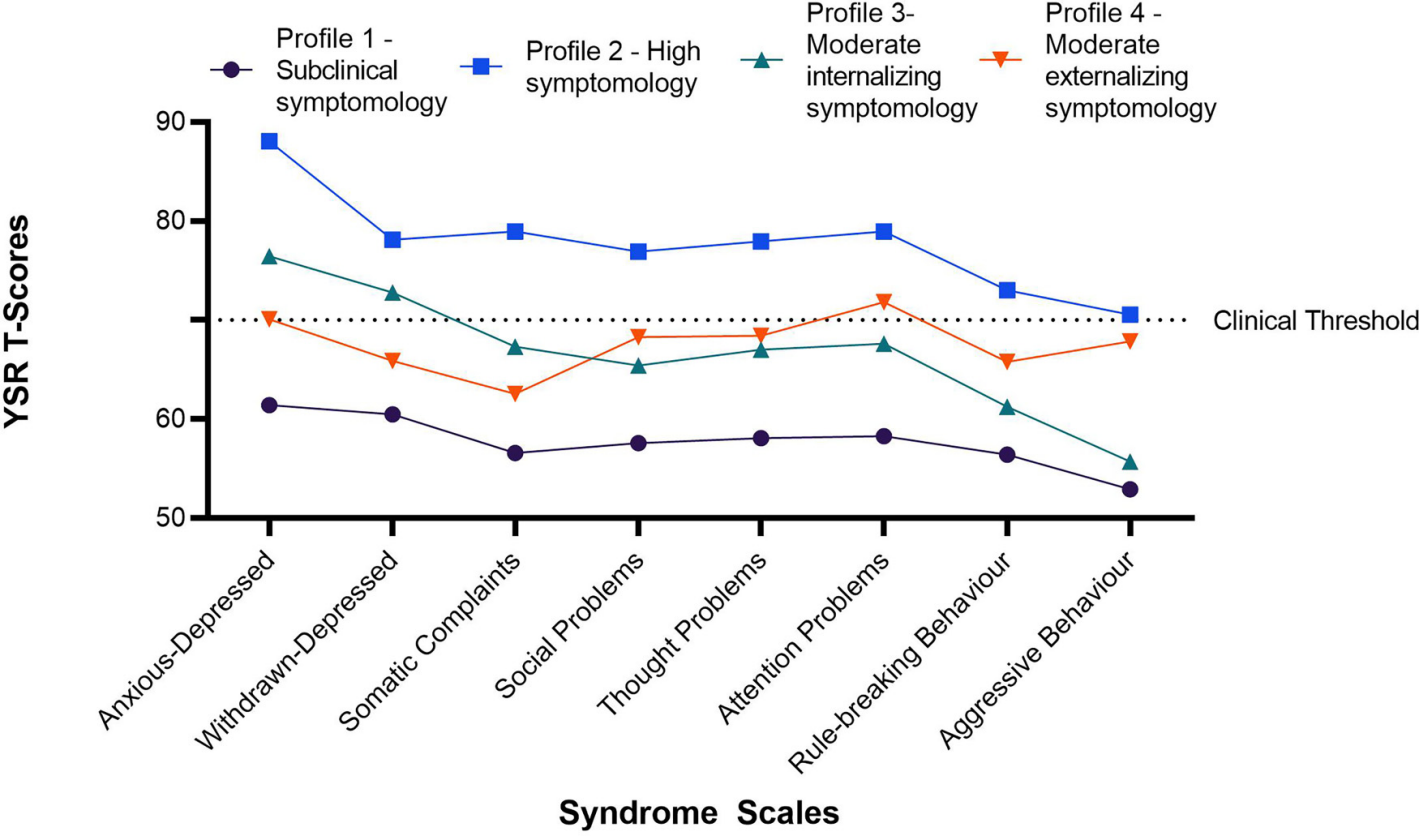
Youth self-report *t*-scores for each symptomology profile.

Figure 1 provides the mean t-scores for the eight syndrome scales according to the four profiles. The first profile named “subclinical symptomology” (38.5% of the sample) had average *t*-scores <70 on each scale. The second profile, “high symptomology” (8.6%), had an average score of ≥70 on all scales. The third profile, “internalizing symptomology” (39.0%), had average *t*-scores ≥70 for anxious-depressed and withdrawn-depressed, and subclinical scores for the remaining scales. The fourth profile, “moderate externalizing symptomology” (13.9%) had average *t*-scores for anxious-depressed and attention problems of ≥70 and subclinical scores for all other scales.

No differences were found in the proportions falling into each profile by organizational type (see Table 3).

**Table 3.** Proportion of Youth Being Treated in Hospital- or Community-Based CAMHS and CAGE-AID, CIS Scores According to Symptom Profiles.

	Proportion of youth within each symptom profile being treated in hospital or community			CAGE-AID scores by symptom profile		CIS scores by symptom profile	
Symptom profile	Hospital	Community	*p* value	Mean score (SD)	*p* value	Mean score (SD)	*p* value
Sub-threshold symptomology	49 (35.8)	41 (41.0)	0.41	0.36 (0.78)	<0.001	14.2 (7.3)	<0.001
High symptomology	6 (4.4)	13 (13.0)	0.016	1.5 (1.7)		33.6 (5.6)	
Moderate internalizing symptomology	61 (44.5)	35 (35.0)	0.14	0.97 (1.4)		22.8 (6.5)	
Moderate externalizing symptomology	21 (15.3)	11 (11.0)	0.336	0.94 (1.4)		23.0 (7.7)	

CAGE-AID = CAGE-adapted to include drugs; CIS = Columbia Impairment Scale; SD = standard deviation; CAMHS = child and adolescent mental health services; LYiTS = Longitudinal Youth in Transition Study.

A third (33.3%, *n* = 79) of the sample screened as being at high risk for alcohol or drug problem behaviours, with no difference between those getting care in the community (38.0%, *n* = 38) and in hospital (29.9%, *n* = 41; *p* = 0.19).

CIS scores were not different between those receiving community (19.9; SD = 8.3) or hospital (21.2; SD = 9.6; *p* = 0.25) care. About two-thirds of those treated in hospital (67.9%) and community (72.0%) scored above the cut off indicating clinically impaired levels of functioning; the difference was not significant (*p* = 0.50).

Mean CIS and CAGE scores varied significantly across the four profiles identified using the YSR syndrome scores in expected ways (see Table 3).

The five most prevalent health conditions were acne (29.9%), developmental disorder (25.4%), allergies (23.8%), migraine/headache (23.0%), sleep disturbance/problems (20.9%). No difference was found by organization type for any listed condition. Two-thirds reported two or more conditions (63.3%), with no difference in multimorbidity by organization type (*p* = 0.37).

More treated in the community reported having a family doctor (93%) compared to youth in hospital-based care (80%, *p* = 0.005). Psychiatrist use also varied by organization type. More of those receiving hospital-based care reported a psychiatrist as their primary care provider (77%) than those in community-based care (10%, p < 0.001, See Table 4). Most of those in hospital care reported seeing a psychiatrist in the past 6 months (93%), with a median of 4 visits (IQR = 2–8), compared to about half of those receiving community-based care (55%) and a median of 1 visit (IQR = 0–4). More of those receiving hospital- than community-based care reported medication use (75% vs. 57%, *p* = 0.005).

**Table 4.** Health Care Utilization Among the LYiTS Cohort and Comparison by Hospital- Versus Community- Based CAMHS.

	*N* (%)				Median visits/admissions (IQR)			
	Overall *N* = 237	Hospital *N* = 137	Community *N* = 100	*p* value	Overall	Hospital	Community	*p* value
Has a family doctor	200/234	107/134 (79.9)	93/100 (93.0%)	0.005				
Psychiatrist reported as primary care provider	113/229 (49.3)	104/135 (77.0)	9/94 (9.6)	<0.001	–	–	–	–
Psychiatrist use	183/237 (77.2)	128/137 (93.4)	55/100 (55.0)	<0.001	3 (0–7)	4 (2–8)	1 (0–4)	<0.001
Alternative mental health care provider use^a^	206/237 (86.9)	110/137 (80.3)	96/100 (96.0)	<0.001	12 (3–25)	8 (2–20)	20.5 (12–33)	<0.001
Emergency department use	66/237 (28.0)	38/137 (27.7)	28/100 (28.0)	0.964	1 (1–2)	1 (1–2)	1 (1–2)	0.553
Hospital admissions	39/236 (16.6)	22/137 (16.1)	17/99 (17.4)	0.82	1 (1–1)	1 (1–1)	1 (1–2)	0.032
Days in hospital	–	–	–	–	3 (1–7)	1 (1–3.5)	5 (2–11)	0.011
Medication use in last 2 days	159/237 (67.1)	102/137 (74.5)	57/100 (57.0)	0.005	–	–	–	–

CAMHS = child and adolescent mental health services; IQR = interquartile range; LYiTS = Longitudinal Youth in Transition Study

^a^ Includes social workers, psychologists, child and youth workers, and nurses.

Significantly more of those receiving community-based care saw an allied health services provider in the past 6 months than those in hospital-based care (96% vs. 80%, *p* < 0.001) and had 2.5 times more visits to this provider type (*p* < 0.001).

## Discussion

Recommended stepped-care service delivery models suggest high-intensity interventions, such as pharmacological treatment, should be implemented only for youth with more intensive symptom management needs.^32^ Accordingly, we hypothesized that youth receiving hospital-based care, where there is a higher density of medical specialists, would report a higher symptom burden. Against expectations, we found no differences in psychiatric symptoms (mean levels or proportion with clinically elevated score), substance use or functioning. Moreover, there was no difference in the proportion falling into each of the four YSR symptomology profiles identified. These findings underscore the need for further research examining factors leading to receiving care in different service types, particularly as there is evidence that demographic or socio-economic factors such as sex and income may predict service use patterns in addition to clinical factors.^21^ Further, average wait times for CAMHS in Ontario are consistently over two months, with some regions stretching up to 2 years.^4^ Given lengthy wait times and lack of centralized intake across CAMHS sectors, youth often seek care from multiple agencies simultaneously,^22^ potentially resulting in youth receiving treatment where they can first access it, rather than where it is most clinically appropriate.

Despite the similarity in symptom burden and functioning to youth receiving community-based care, youth receiving hospital-based care more often reported a psychiatrist as their primary provider and any psychiatrist involvement in their care. This may reflect the staffing models – the community sites each have 4–5 psychiatrists on staff, including a number working part-time hours, while each hospital site has approximately 18–22 psychiatrists on staff. Hospital-based participants also reported a higher frequency of medication use, which may relate to more psychiatrist-led care in this setting. Less than a third of psychiatrists in Ontario spend a significant amount of their clinical practice on psychotherapy services,^36^ meaning psychiatrists may be more focussed on providing services to youth who require medication management as a core part of their care. Since time in care was similar, it is possible that more frequent medication use in the hospital-based participants could be masking clinical differences between the organizational sites, resulting in similarities in symptom burden found in this study.

Though the frequency of medication use was lower in the community, and despite reporting minimal psychiatrist involvement, over half of the youth receiving community-based services reported taking medication. Further, a larger proportion of community-based youth was accessing care from a family doctor. General practitioners, who are the primary care providers for most transition-age youth,^14^ may be managing medications for youth receiving community-based services from allied health providers. This aligns with recent reports that general practitioners are increasingly prescribing and managing antidepressants in adolescents,^37^ often without the involvement of a mental health specialist.^38^

The hospital- and community-based participants reported similar frequency of ED use and inpatient admissions within the past 6 months. This could, alongside the similar symptom profiles, suggest that the hospital- and community-based programmes are serving youth with a similar level of acuity, or that connection to either type of service has a similar effect on adolescents’ need for crisis services. Ongoing connection to community or outpatient CAMHS has been shown to be accompanied by reduced use of acute care and crisis services,^21^ although there is also conflicting evidence suggesting that these programmes have minimal impact on ED visits for mental health.^39^

Four-profile symptom models have been identified previously in pre-adolescents from the general population using the parent-report version of the YSR,^40^ and in adolescents recruited from inpatient mental health services using the YSR.^41^ In the latter sample, Berona et al.^41^ identified four profiles with very similar symptom clusters found in our study, providing validity to our model along with the finding that CIS and CAGE scores vary across the profile types in expected ways. Further examination of this type of model and treatment outcomes could provide information about the intensity of interventions best suited across different symptom profiles^42^ and inform triaging and clinical decision-making in CAMHS.

In our sample and Berona et al.’s,^41^ over one-third of the youth fell into a subclinical/low YSR symptomology profile. As subthreshold symptoms can be accompanied by significant functional impairment,^43^ profiles should be considered in conjunction with other indicators, including the level of impairment or previous health care utilization, when assessing clinical stage and formulating treatment intensity.^44–46^ In the LYiTS sample, the mean CIS score for the subclinical symptomology group fell slightly below the cut-off score for clinically significant impairment. These youth may thus potentially benefit from timely, less intensive interventions delivered within primary care or community-based models, which are increasingly supported as effective and accessible means of delivering mental health care.^47^

This analysis of the LYiTS cohort baseline has several strengths. It is, to our knowledge, one of the first analyses of a clinically derived sample from both hospital- and community-based CAMHS. The LYiTS study was co-designed with youth with lived experience, which likely contributed to the high response rate and minimal missing data. Finally, the use of detailed self-report measures allowed us to capture the full breadth of mental health services use, including care provided by non-physician providers whose services are not captured in Ontario’s administrative data sets.

This study is not without limitations. While this is a multi-site study, youth were recruited within a single large Canadian city with a high density of specialized mental health care providers,^48^ potentially limiting the relevance for populations from more rural areas with less access to CAMHS. The community sites in this study, while staffed with significantly fewer psychiatrists than the hospital sites, still have access to staff psychiatrists and trainees through their academic affiliation, meaning that the model of care may differ somewhat from other community CAMHS with minimal or no access to on-staff psychiatrists. Further research is needed to understand whether the similarities found within our sample hold when including community CAMHS without psychiatrists. Moreover, without access to administrative data, we are unable to determine how representative our sample was of youth receiving care in the participating centres, at any particular time. While the overall response rate was about 55%, this proportion was higher in the community settings. This may have resulted in the hospital sample being more highly selected and less representative than the community sample, which may have implications for the comparisons undertaken. Further, results may not reflect the experience of youth who are not able to read and write in English or who experience limitations in their cognitive functioning. Also since youth were not randomized to receive care in the hospital versus community, there could be uncaptured characteristics leading youth to access care in a particular type of setting and contributing to their reported symptom profiles. Finally, we did not have objective indicators of acuity or functioning, such as clinician-reported diagnoses or school records reflecting absenteeism and grades. The latter measures would provide insight into impairment to developmentally appropriate participation. Further youth engagement will be important for identifying other relevant measures of impact. Future research linking administrative health care data will be critical to determining whether health service utilization trajectories prior to CAMHS intake differed between the hospital and community samples and whether these trajectories differ by illness onset and severity.

## Conclusion

Transition-aged youth face both elevated risks for new onset mental health challenges and complex pathways into and within mental health care systems. We found no differences in psychiatric symptom burden between youth at community versus hospital sites. Despite this, there were differences in use of psychiatrist-led care and medication, raising questions for ongoing research about what determines access to different levels of care for youth. This analysis provides important context for better understanding TAY as they cross the transition boundary, as differing health care access prior to the transition boundary may impact their access to care as they age out of CAMHS.

## Supplemental Material

sj-docx-1-cpa-10.1177_07067437221115947 - Supplemental material for The Longitudinal Youth in Transition Study (LYiTS) Cohort Profile: Exploration by Hospital- Versus Community-Based Mental Health Services Supplemental material, sj-docx-1-cpa-10.1177_07067437221115947 for The Longitudinal Youth in Transition Study (LYiTS) Cohort Profile: Exploration by Hospital- Versus Community-Based Mental Health Services by Kristin Cleverley, Julia Davies, Sarah Brennenstuhl, Kathryn J. Bennett, Amy Cheung, Joanna Henderson, Daphne J. Korczak, Paul Kurdyak, Andrea Levinson, Antonio Pignatiello, Katye Stevens, Aristotle N. Voineskos and Peter Szatmari in The Canadian Journal of Psychiatry
