# Supplementary material for: The Longitudinal Youth in Transition Study (LYiTS) Cohort Profile: Exploration by Hospital- Versus Community-Based Mental Health Services
Source: Can J Psychiatry. 2022 Aug 4;67(12):928–38. doi: 10.1177/07067437221115947 (PMC9659798; doi:10.1177/07067437221115947)
Supplement: sj-docx-1-cpa-10.1177_07067437221115947 - Supplemental material for The Longitudinal Youth in Transition Study (LYiTS) Cohort Profile: Exploration by Hospital- Versus Community-Based Mental Health Services [file sj-docx-1-cpa-10.1177_07067437221115947.docx]

**Supplemental File**

***Latent Profile Analysis Methods used.***

Using Mplus (v7), models with increasing number of profiles were estimated until size of group membership in any one became too small for interpretability. The following model fit criteria guided optimal model selection: 1) lower relative values on Akaike’s Information Criterion (AIC), Bayesian Information Criterion (BIC) and sample-sized adjusted BIC, 2) significant bootstrap likelihood ratio test (BLRT) and Lo-Mendell-Rubin Adjusted Likelihood Ratio test comparing models with adjacent number of classes, and 3) higher relative entropy values indicating better classification accuracy^32^. Models were estimated using maximum likelihood with robust standard errors and converged on a replicated solution of the best likelihood value to avoid local maxima^33^. Using the most likely class variable, for each profile, proportions of those treated in the community and hospital were compared using a chi square test. Literature suggests that a sample of 200+ should provide ample power for the BLRT, based on a 4-class solution, with moderate separate and 8 indicators.^34^

***Details of Latent Profile Analysis Results.***

Models of one to five profiles were estimated. Models with more than five profiles were rejected because the class with the lowest sample proportion became too small to be reliably estimated (1.3%). The BLRT value was significant for all estimated models. Relative values of BIC, AIC, and SABIC declined with models of increasing numbers of profiles up to the model with four profiles, after which the decline stopped being meaningful. The highest entropy value was found for the model with 4 profiles.

| **Table. Comparison of LCA models with Different Numbers of Classes According to Selection Criteria** | | | | | | | | |  |
| --- | --- | --- | --- | --- | --- | --- | --- | --- | --- |
|  |  |  |  |  |  |  |  |  |  |
|  | BIC | AIC | Sample-size Adjusted BIC | Adjusted LMR test | BLRT | Entropy |  |  |  |
| 1 class | 13811.449 | 13755.96 | 13760.735 | --- | --- | 1 |  |  |  |
| 2 classes | 13422.286 | 13335.585 | 13343.045 | <.001 | <.001 | 0.815 |  |  |  |
| 3 classes | 13309.267 | 13191.353 | 13201.499 | 0.118 | <.001 | 0.844 |  |  |  |
| 4 classes | 13272.623 | 13123.497 | 13136.328 | 0.101 | <.001 | 0.862 |  |  |  |
| 5 classes | 13265.654 | 13085.315 | 13100.833 | 0.168 | <.001 | 0.848 |  |  |  |
| BIC, Bayesian Information Criterion; AIC, Akaike’s Information Criterion LMR, Lo-Mendell-Rubin; BLRT, bootstrap likelihood ratio test | | | | | | | | |  |
|  |  |  |  |  |  |  |  |  |  |
